# Protective Effect of Vitamin D against Hepatic Molecular Apoptosis Caused by a High-Fat Diet in Rats

**DOI:** 10.3390/cimb45010031

**Published:** 2023-01-05

**Authors:** Huda F. Alshaibi, Sherin Bakhashab, Asma Almuhammadi, Yusuf S. Althobaiti, Mohammed A. Baghdadi, Khadeejah Alsolami

**Affiliations:** 1 Department of Biochemistry, Faculty of Sciences, King Abdulaziz University, Jeddah 21589, Saudi Arabia; 2 Embryonic Stem Cell Unit, King Fahd Medical Research Center, King Abdulaziz University, Jeddah 21589, Saudi Arabia; 3 Center of Excellence in Genomic Medicine Research, King Abdulaziz University, Jeddah 21589, Saudi Arabia; 4 Department of Biological Sciences, Faculty of Sciences, King Abdulaziz University, Jeddah 21589, Saudi Arabia; 5 Biology Department, College of Science, Jouf University, P.O. Box 2014, Sakaka 72388, Saudi Arabia; 6 Department of Pharmacology and Toxicology, College of Pharmacy, Taif University, P.O. Box 11099, Taif 21944, Saudi Arabia; 7 Addiction and Neuroscience Research Unit, Taif University, Taif 21944, Saudi Arabia; 8 Research Center, King Faisal Specialist Hospital and Research Center, Jeddah 21589, Saudi Arabia

**Keywords:** high-fat diet, vitamin D, hepatic apoptosis, inflammatory cytokine, anti-apoptosis

## Abstract

The protective effects of vitamin D (VitD) in different diseases were studied. The liver is of great interest, especially with the presence of VitD receptors. A high-fat diet (HFD) is associated with many diseases, including liver injury. Consumption of saturated fatty acids triggers hepatic apoptosis and is associated with increased inflammation. We aimed in this study to investigate the protective effects of VitD on hepatic molecular apoptotic changes in response to an HFD in rats. Forty male Wistar albino rats were used and divided into four groups: control, HFD, control + VitD, and VitD-supplemented HFD (HFD + VitD) groups. After six months, the rats were sacrificed, and the livers were removed. RNA was extracted from liver tissues and used for the quantitative real-time RT-PCR of different genes: B-cell lymphoma/leukemia-2 (BCL2), BCL-2-associated X protein (Bax), Fas cell surface death receptor (FAS), FAS ligand (FASL), and tumor necrosis factor α (TNF-α). The results showed that an HFD increased the expression of the pro-apoptotic genes Bax, FAS, and FASL, and reduced the expression of the anti-apoptotic gene BCL2. Interestingly, a VitD-supplemented HFD significantly increased the BCL2 expression and decreased the expression of all pro-apoptotic genes and TNFα. In conclusion, VitD has a protective role against hepatic molecular apoptotic changes in response to an HFD.

## 1. Introduction

Liver damage can be triggered by different factors, such as alcohol intake, viral infection, drug abuse, and the consumption of a fat-rich diet, particularly saturated fatty acids [1,2,3,4]. Saturated fatty acids accumulate in hepatocytes, resulting in cell death via various death modes, including apoptosis, necrosis, and necroptosis [5,6,7].

Apoptosis primarily occurs through two main mechanisms that involve either the internal mitochondrial intrinsic pathway or the external death receptor extrinsic pathway [8]. The pro-apoptotic protein B-cell lymphoma/leukemia-2 (*Bcl2*)-associated X protein (*Bax*) mediates the mitochondrial intrinsic pathway by a series of cascade signal transduction pathways that end by activating caspase-9, which is the promoter of the intrinsic pathway. In contrast, *Bcl2* acts as an anti-apoptotic protein in this process [9]. The death receptor pathway is mediated by various proteins that belong to the tumor necrosis factor (TNF) superfamily, including *FAS* and TNF receptor type 1 (TNFR1) [10]. Once *TNF-α* binds to its receptor, it recruits the TNFR1-associated death domain (TRADD) adapter protein [10]. This activation of TNFR1 triggers the formation of several signaling complexes called Complex I, IIa, IIb, and IIc, and each one of these complexes is responsible for specific cellular responses [11,12]. Complex I, for example, results in the activation of nuclear factor _k_B (NF-_k_B) and mitogen-activated protein kinases (MAPKs), thus resulting in inducing inflammation, cellular proliferation, and cellular survival [12,13,14,15]. Complex IIa and Complex IIb activate caspase-8, which is the promoter of the extrinsic apoptosis pathway [9,16]. Complex IIc consists of receptor-interacting serine/threonine protein kinase 1 (RIPK1) and receptor-interacting serine/threonine protein kinase 3 (RIPK3). This complex activates the mixed lineage kinase domain-like protein (MLKL). The outcome of this signaling cascade is the induction of necroptosis and inflammation [16,17]. In addition, *TNF-α* acts as an important inflammatory cytokine synthesized in response to the presence of reactive oxygen species and impaired *α*-oxidation enzymes due to the consumption of a high-fat diet (HFD) [18,19,20]. Thus, it can cause both hepatocyte apoptosis and necroptosis, which may result in chronic liver inflammation, creating a vicious cycle [19,21].

Vitamin D (VitD) was previously mainly linked with calcium/phosphorus homeostasis, bone health, and growth. However, in the last few decades, VitD has become known to be associated with various cellular functions, such as cellular proliferation, differentiation, immunomodulation, and apoptosis [22,23]. Therefore, many studies have focused on the protective role of VitD in several diseases, including hypertension, diabetes, cardiovascular disease, and many more [22,24,25,26]. The role of VitD in hepatic pathophysiology and its progression has attracted attention after studies have reported the upregulation of VitD receptors (VDR) in injured hepatocytes such as hepatocellular carcinoma and nonalcoholic fatty liver disease (NAFLD) compared with normal hepatocytes [27,28,29,30].

However, studies on this topic remain limited. Therefore, this study aims to investigate the protective role of VitD on hepatic molecular apoptotic changes in response to an HFD in rats.

## 2. Materials and Methods

### 2.1. Animals

Male Wistar albino rats (*n* = 40; weighing 150–200 g) were used in this study. The rats were obtained from King Fahad Medical Research Centre, Jeddah, Saudi Arabia, and kept in the animal house unit during the time of the study. The rats were housed under a standard laboratory temperature (23 °C ± 3 °C) and humidity, and a natural 12 h:12 h light/dark cycle, with free access to water ad libitum. All animals were kept under conditions that prevented them from experiencing unnecessary pain and discomfort according to the standard guidelines of the European College of Laboratory Animal Medicine. The study was approved by the Ethical Committee of the Faculty of Medicine, King Abdulaziz University, Jeddah, Saudi Arabia (reference number 369-16).

### 2.2. Diets and VitD

A standard diet and an HFD were acquired from Research Diets Inc., New Brunswick, NJ, USA, and the composition of both diets is listed in Table 1. VitD was purchased from Sigma-Aldrich Co., St. Louis, MO, USA.

### 2.3. Experimental Design

After 1 week of acclimatization, the rats were randomly divided into the following groups:

Group I (control; C): rats in this group received the standard diet for 6 months (*n* = 10).

Group II (control + VitD): rats in this group received the standard diet for six months and were co-administered with vitamin D by gavage in a dose of 400 IU/kg/day for six months (*n* = 10).

Group II (HFD): rats in this group received an HFD for 6 months (*n* = 10).

Group III (HFD + VitD): rats in this group received the HFD for 6 months and were co-administered with VitD by oral gavage in a dose of 400 IU/kg/day [31,32] (*n* = 10).

Food intake was monitored throughout the study. Body weight and body length (oral to anus length, OA) were measured at the beginning to assess body mass index (BMI) (body weight [g] / the square of OA length [cm^2^]). This procedure was repeated every 45 days and at the end of the experiment [33]. After 6 months, the rats were sacrificed under anesthesia using diethyl ether. The livers were removed and washed with normal saline. RNA was preserved for quantitative real-time polymerase chain reaction (qRT-PCR) assessment.

### 2.4. qRT-PCR

Total RNA was extracted from liver tissues using an RNAeasy Mini Kit (Qiagen, Hilden, Germany) according to the manufacturer’s instructions. The *FAS* and *FAS* ligand (*FASL*) genes and the *Bax*, *Bcl2*, and *TNFα* genes were selected for the extrinsic and intrinsic apoptotic pathways, respectively.

Total RNA (5 μg) was reverse transcribed into cDNA in a final reaction mix of 20 μL using a reverse kit (ImProm-IITm Reverse Transcription System, Promega, Madison, WI, USA, cat no. A3800), according to the manufacturer’s instructions. The reaction was conducted on a thermal cycler with the following cycling conditions: 25 °C for 5 min, 42 °C for 120 min, and 70 °C for 15 min.

qRT-PCR was performed on a StepOne plus Real-Time PCR system (Thermo Fisher Scientific, Waltham, MA, USA) in a 20 μL reaction mix containing 2 μL of cDNA, 10 μL of EverGreen Universal qPCR Master Mix (2X) (Haven Scientific, Jeddah, Saudi Arabia), 6 μL of DNase/RNase-free water (Thermo Fisher Scientific), and 1 μL of each forward/reverse primer of the target and reference genes (Macrogen, Seoul, Republic of Korea). The list of primer genes is shown in Table 2. Thermocycling was conducted at 95 °C for 2 min, followed by 40 cycles at 95 °C for 15 s, and 60 °C for 1 min. All samples were performed in triplicate. The relative gene expression of each target gene was quantified using the 2^−ΔΔCT^ method, which was normalized to the reference gene for glyceraldehyde-3-phosphate dehydrogenase (GAPDH). The fold change was calculated using the equation ∆dct/3.3 × −10, ∆dct/3.3 ×−10 [34].

### 2.5. Statistical Analyses

The data were processed using GraphPad Prism 9 (GraphPad Software, Inc., La Jolla, CA, USA), and the results were presented as the mean ± SEM. Statistical significance was tested using one-way analysis of variance (ANOVA) and Šídák’s multiple comparison test to identify the significant differences between groups. A *p*-value of <0.05 was considered significant.

## 3. Results

### 3.1. BMI of HFD and HFD + VitD Groups

At the end of the 6 months, the body weight, OA length, and BMI were not significantly different compared with the controls, as shown in Table 3.

### 3.2. qRT-PCR

#### 3.2.1. Intrinsic Apoptotic Pathway Genes

**Figure 1:**
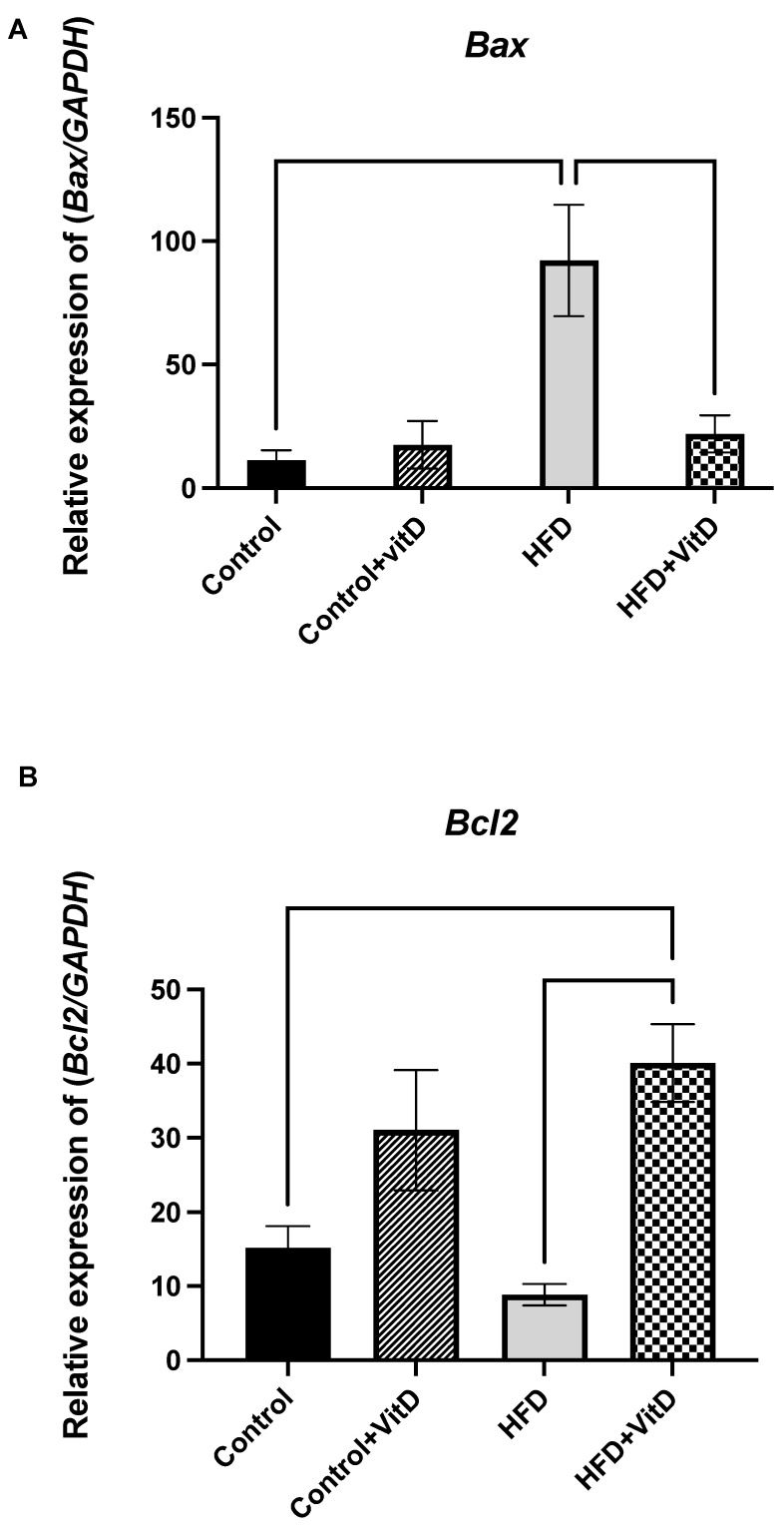
Gene expression of the intrinsic apoptotic signaling genes. (**A**) The gene expression of the pro-apoptotic *Bax* in rats fed with a normal diet (Control), a high-fat diet (HFD), and a vitamin D-supplemented HFD (HFD + VitD). Bax was significantly increased in the group fed with the HFD (*p* = 0.013) relative to the control. Combining vitamin D with the HFD downregulated the expression of this gene (*p* = 0.02). (**B**) The gene expression of the anti-apoptotic *Bcl2*. Combining VitD with the HFD caused a significant upregulation relative to the control and HFD groups (*p* = 0.0056, *p* ≤ 0.0001). Data were normalized to the reference gene GAPDH. All data were expressed as mean ± SEM. Data were considered significant if *p* < 0.05.

There was no significant difference between the control group and the control group supplemented with VitD in the expression of both *Bax* and *Bcl2*. Gene expression of the intrinsic apoptotic signaling gene pro-apoptotic *Bax* and the anti-apoptotic gene *Bcl2* were measured. At the end of the six months, the HFD significantly increased the expression of the pro-apoptotic gene *Bax* relative to the control (9.7-fold change, *p* = 0.013, one-way ANOVA, Šídák’s test). In contrast, the HFD decreased the expression of the anti-apoptotic gene *Bcl2* (−4.2-fold change); however, this reduction was not significant. The rats given a combination of VitD and HFD exhibited a significant downregulation of the *Bax* gene toward the normal level (−1-fold change, *p* = 0.02), whereas *Bcl2* was significantly upregulated (2.2-fold change, *p* ≤ 0.0001, one-way ANOVA, Šídák’s test), as shown in Figure 1A,B.

#### 3.2.2. Extrinsic Apoptotic Pathway Genes

**Figure 2:**
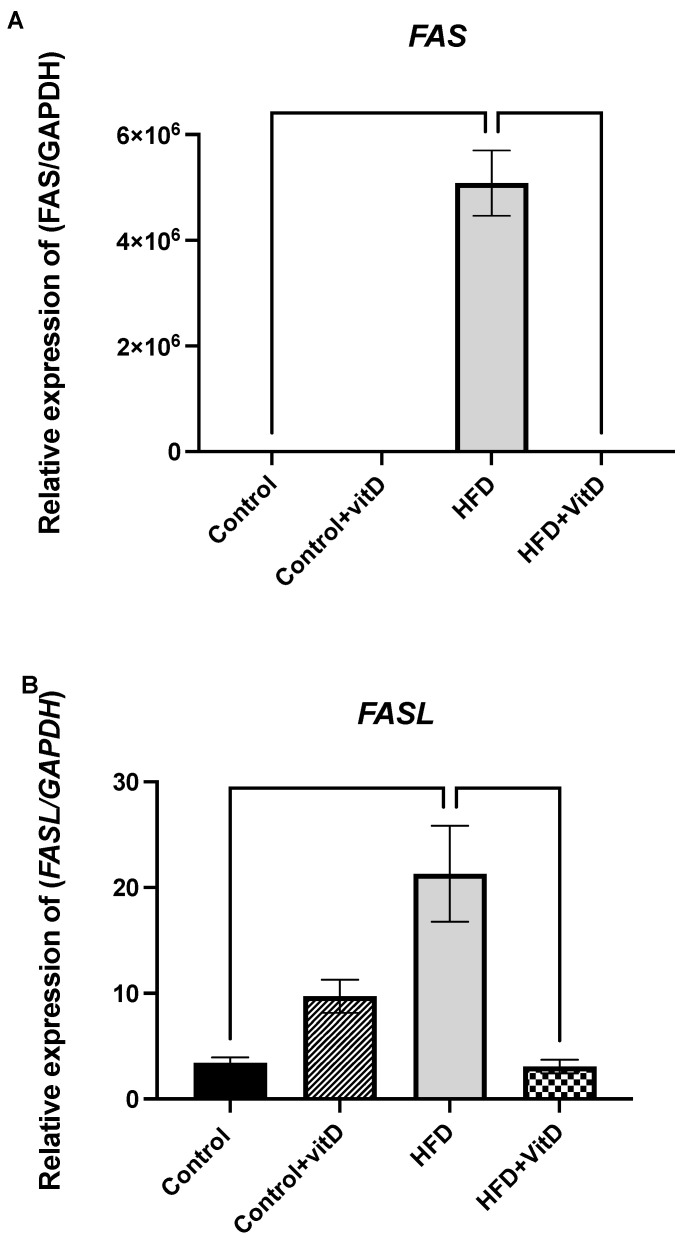
Gene expression of the extrinsic apoptotic signaling genes. (**A**) The gene expression of *FAS* in rats fed a normal diet (Control), a high-fat diet (HFD), and a vitamin D-supplemented HFD (HFD + VitD). *FAS* was increased in the group fed with HFD (*p* ≤ 0.0001) compared with the control. Combining vitamin D with HFD caused this gene to be significantly downregulated (*p* ≤ 0.0001). (**B**) The gene expression of FAS ligand (FASL). The HFD increased the expression of *FASL* in comparison to the control (*p* ≤ 0.0001). Combining vitamin D with the HFD significantly downregulated this gene in comparison to the control (*p* ≤ 0.0001). Data were normalized to the reference gene GAPDH. All data were expressed as mean ± SEM. Data were considered significant if *p* < 0.05.

There was no significant difference between the control group and the control group supplemented with VitD in the expression of both *FAS* and *FASL*. Gene expression of the extrinsic apoptotic signaling genes *FAS* and *FASL* were measured. At the end of the six months, the HFD significantly increased the expression of both *FAS* and *FASL* genes relative to the control, with 63.4- and 6.9-fold changes, respectively (*p* ≤ 0.0001, *p* ≤ 0.0001, one-way ANOVA, Šídák’s test). However, in the HFD + VitD rats’ group, the expression of the *FAS* and *FASL* genes was at normal levels and significantly different relative to the rats in the HFD group, with −2.5- and −2.2-fold changes, respectively (*p* ≤ 0.0001, *p* ≤ 0.0001, one-way ANOVA, Šídák’s test) (Figure 2A,B).

### 3.3. TNF-α as an Inflammatory and Apoptotic Mediator

**Figure 3:**
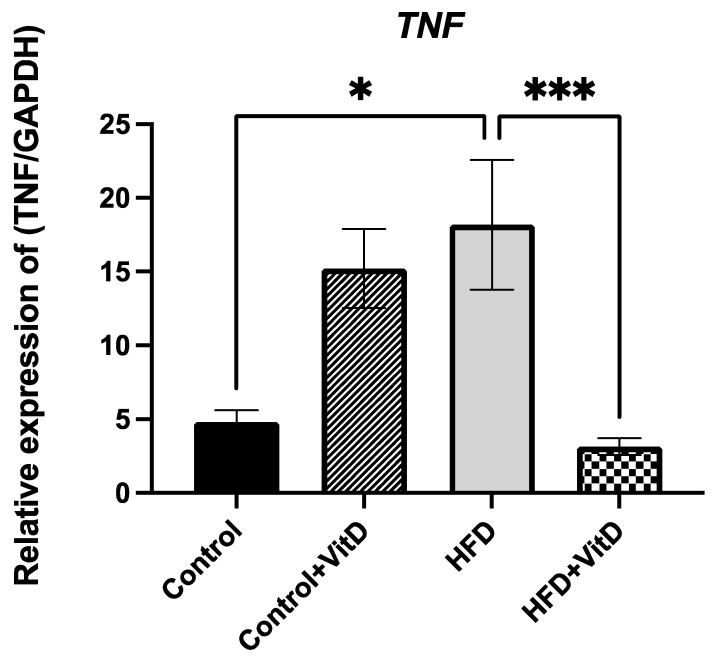
Tumor necrosis factor (*TNF-α*) gene expression. The high-fat diet (HFD) increased the expression of *TNFα* significantly (*p* = 0.03) compared to the control, whereas VitD supplementation with the HFD significantly decreased the expression of *TNFα* (*p* = 0.0006). Data were normalized to the reference gene GAPDH. All data were expressed as mean ± SEM. Data were considered significant if * *p* < 0.05, *** *p* < 0.001.

After six months, there was no significant difference between the control group and the control group supplemented with VitD in the expression of *TNF-α,* while the expression of *TNF-α* increased in rats in the HFD group significantly (1.8-fold change *p* = 0.03, one-way ANOVA, Šídák’s test). In contrast, in the HFD + VitD group, the expression was significantly downregulated relative to the group fed with the HFD (−2.7-fold change, *p* = 0.0006, one-way ANOVA, Šídák’s test), as shown in Figure 3.

## 4. Discussion

Chronic consumption of an HFD, particularly saturated fatty acids, is strongly associated with hepatocyte apoptosis [35]. Both the intrinsic mitochondrial pathway and the extrinsic death receptor pathway are stimulated by an HFD. The present study showed the protective effect of VitD against molecular apoptotic changes in rats fed an HFD for 6 months.

No significant changes were found in body weight and BMI between HFD- (45% fat) and HFD + VitD-fed rats relative to the control group. These results were similar to the study carried out by Ramalho et al. 2017, suggesting that an HFD is linked to hyperinsulinemia and insulin resistance without developing obesity [36].

In this study, we showed that an HFD stimulated the intrinsic apoptosis pathway of hepatocytes, indicated by the significant increase in the gene expression of the pro-apoptotic gene *Bax* and the decreased gene expression of the anti-apoptotic gene *Bcl2* relative to the control group. Other studies reported the redistribution of Cathepsin B into the cytoplasm by enhancing the translocation of *Bax* to lysosomes in response to an HFD [37,38]. In addition, free fatty acids increase lysosomal permeabilization, which may lead to mitochondrial dysfunction, thus demonstrating the role of exogenous fat on lysosomes in the initiation of the intrinsic apoptotic pathway [38,39]. An HFD supplemented with VitD decreased the gene expression of *Bax* and increased the gene expression of *Bcl2* back to a normal level compared to the control. These results suggest a protective effect of VitD in an HFD. The same protective effect of VitD has been previously reported [40]. In addition, a recent study has investigated the role of VitD in non-alcoholic fatty liver disease in rats and reported a similar result found in our study: VitD injection enhances the expression of *Bcl2* compared to the group fed only with an HFD [41].

The harmful effect of the HFD on triggering apoptosis was not only limited to the activation of the intrinsic pathway, as mentioned earlier, but was also involved in activating the extrinsic pathway. This finding was based on the significant increase in both *FAS* and *FASL* gene expression in the HFD-fed rats. This increase in gene expression was significantly reduced in the group receiving the VitD-supplemented HFD. To our knowledge, the protective effect of VitD on the extrinsic apoptotic genes *FAS* and *FASL* was not investigated in the liver before. However, several studies have reported the same protective effect of VitD against pathological changes in extrinsic gene expression pathways in different organs, including the heart and spleen [31,42,43,44].

Feeding rats with an HFD not only induced the gene expression of intrinsic and extrinsic apoptosis but also increased the hepatic expression of the inflammatory cytokine *TNF-α* compared to the control. Thus, increased apoptosis could be one of the suggested mechanisms that can cause liver inflammation and vice versa. The expressions of both *FAS* and *TNF-α* are elevated in liver biopsy samples collected from NAFL patients and in hepatocyte cell lines treated with free fatty acids [45,46]. Accordingly, hepatocyte apoptosis is thoroughly associated with hepatic inflammation and fibrosis [47,48]. We found that VitD significantly reduced the gene expression of *TNF-α* compared to the HFD-fed group, which is consistent with several other studies that reported the anti-inflammatory activity of VitD [40,41,49,50]. However, the protective effect of VitD was only associated with the HFD but had no protective role in the normal control group since there were no differences between the two control groups in all studied genes.

Several suggested mechanisms may explain the protective role of VitD against inflammation and apoptosis, and one of these mechanisms is through binding with specific VDR, as reported previously [38]. Another suggested mechanism is that VitD downregulates the toll-like receptor 4-mediated inflammatory pathway, which may be activated by an HFD and fatty acid deposition in the liver [51,52].

## 5. Conclusions

Our results showed that feeding rats an HFD for 6 months increased both intrinsic and extrinsic apoptotic gene expressions; however, body weight was not increased. In addition, it increased the inflammatory cytokine *TNF-α*, which may also induce the shared intracellular pathway between apoptosis and necrosis, called necroptosis. VitD supplementation was effective in inhibiting apoptosis and inflammation by reducing the expression of *Bax*, *FAS*, *FASL*, and *TNF-α*, as well as decreasing the expression of *Bcl2*. The limitation of this study was the lack of funds. However, future studies are required where more apoptotic genes may be studied, as well as investigating the protective role of VitD in reducing hepatic apoptosis and inflammation in response to an HFD at the cellular level.

## Figures and Tables

**Table 1:** Standard diet and high-fat diet (HFD) composition.

	Standard Diet, D12450H		HFD D12451	
Product Details	gm%	Kcl%	gm%	Kcl%
Protein	19.2	20	24	20
Carbohydrate	67.3	70	41	35
Fat	4.3	10	24	45
Total	-	100	-	100
Kcl/gm	3.58	-	4.73	-

**Table 2:** Rat primers of all targeted genes used in the quantitative real-time polymerase chain reaction.

Rat Primers	Forward Primer	Reverse Primer
*FAS*	CTGATAGCATCTCTGAGG	CTGATAGCATCTCTGAGG
*FASL*	GACAACATAGAGCTGTGG	GACAACATAGAGCTGTGG
*Bax*	CTGGACAACAACATGGAGC	CAGACGGCAACTTCAACTG
*Bcl2*	AGTGGGATACTGGAGATG	CTGGCTGTCTCTGAAGAC
*TNFα*	CTTCTGTCTACTGAACTTCG	CCAATGGCATGGATCTCAA

**Table 3:** Initial and final body weight, oral to anus (OA) length, and body mass index (BMI) after 6 months.

Group	Initial Rat Weight (gm)	Initial AO Length (cm)	BMI (g/cm^2^)	Final Rat Weight (gm)	Final OA Length (cm)	BMI (g/cm^2^)
**Control**	201.4 ± 9.5	20.704 ± 0.8	0.4710 ± 0.04	534.4 ± 42.03	25.5 ± 0.4	0.8218 ± 0.1
**Control + VitD**	215.4 ± 13.4	21.6 ± 0.74	0.4620 ± 0.03	512.4 ± 57.7	25.1 ± 0.42	0.8148 ± 0.1
**HFD**	218.7 ± 7.2	21.45 ± 0.4	0.4755 ± 0.02	521.8 ± 63.42	24.95 ± 0.55	0.8383 ± 0.1
**HFD + VitD**	230.4 ± 12.5	22.26 ± 0.6	0.4649 ± 0.02	534 ± 33.25	25.563 ± 0.5	0.8171 ± 0.04

Control + VitD: rats fed a regular diet treated with vitamin D. HFD: rats fed with a high-fat diet, HFD + VD: rats fed with a high-fat diet treated with vitamin D. Values were expressed as mean ± SD. Data were analyzed using an analysis of variance *t*-test. NS: not significant compared with the BMI value of the control rats.

## Data Availability

Not applicable.
